# A Low-Power Distributed Visual Sensor Network for Real-Time Barcode Localization and Identification

**DOI:** 10.3390/s22041433

**Published:** 2022-02-13

**Authors:** Leander Hendrikx, Rui Zhong, Bruno Cornelis, Adrian Munteanu

**Affiliations:** 1 Electronics and Informatics (ETRO) Department, Vrije Universiteit Brussel (VUB), 1050 Ixelles, Belgium; leanderhendrikx@gmail.com (L.H.); bcorneli@etrovub.be (B.C.); 2 School of Computer, Central China Normal University (CCNU), Wuhan 430079, China; zhongrui0824@126.com

**Keywords:** barcode localization, tracking, object detection, distributed vision network, image sensors

## Abstract

A novel low-power distributed Visual Sensor Network (VSN) system is proposed, which performs real-time collaborative barcode localization, tracking, and robust identification. Due to a dynamic triggering mechanism and efficient transmission protocols, communication is organized amongst the nodes themselves rather than being orchestrated by a single sink node, achieving lower congestion and significantly reducing the vulnerability of the overall system. Specifically, early detection of the moving barcode is achieved through a dynamic triggering mechanism. A hierarchical transmission protocol is designed, within which different communication protocols are used, depending on the type of data exchanged among nodes. Real-Time Transport Protocol (RTP) is employed for video communication, while the Transmission Control Protocol (TCP) and Long Range (LoRa) protocol are used for passing messages amongst the nodes in the VSN. Through an extensive experimental evaluation, we demonstrate that the proposed distributed VSN brings substantial advantages in terms of accuracy, power savings, and time complexity compared to an equivalent system performing centralized processing.

## 1. Introduction

The increased availability of cheap electronics has enabled the construction of low-cost Visual Sensor Network (VSN) platforms that are able to capture, process, and disseminate visual data collectively [1]. A VSN consists of a multitude of small, connected camera sensor nodes, each with their own computation and communication components and power source, which aggregate visual data, process it collaboratively, and transmit useful information to a control center [2]. These platforms provide an excellent solution for many applications, such as video surveillance, personal care, virtual reality, and logistics [3].

This paper proposes a distributed architecture for the real-time localization and identification of multiple barcodes with visual sensor networks. Barcode localization can, for instance, be used for the logistics in big autonomous storage facilities or warehouse management systems, to keep track of robots, objects and personnel.

In contrast to typical centralized architectures, where all the communication must pass through a central sink node [4], a distributed VSN provides communication among the nodes. The distributed VSN paradigm has been proven to be efficient in achieving lower congestion and reducing the vulnerability of the overall system. On the other hand, in distributed VSN, the transmission among nodes causes increased consumption in terms of energy and bandwidth. If only the cameras with informative motion are activated, the overall consumption will be substantially decreased. The triggering mechanism that controls how to activate or deactivate the nodes is a critical factor affecting the overall energy and bandwidth consumption. However, the prior art in distributed VSN failed to propose an appropriate triggering mechanism. The triggering mechanisms in current distributed VSNs are barely designed in a distributed fashion, such as the triggering method based on clustering informative observation [5]. In [6], the prediction of a node’s state is performed via the fusion of states passed from multiple nodes to a sink node.

In this work, we propose a dynamic triggering mechanism operating a distributed fashion, where a wake-up message is sent from neighboring activated cameras to trigger the activation of the relevant neighboring cameras. In addition, prior work [5,7] tends to transmit multiple types of data via a single Ethernet protocol. Since the transmitted packages combine multiple types of data, package loss could cause severe problems for the target tracking in a distributed VSN. Thus, a hierarchical transmission protocol is also presented in our work to mitigate the damage of package loss. The fundamental idea is to schedule the data transmission depending on the type of data exchanged among nodes. This approach enhances the robustness of the system via the hierarchical transmission protocol.

**Figure 1:**
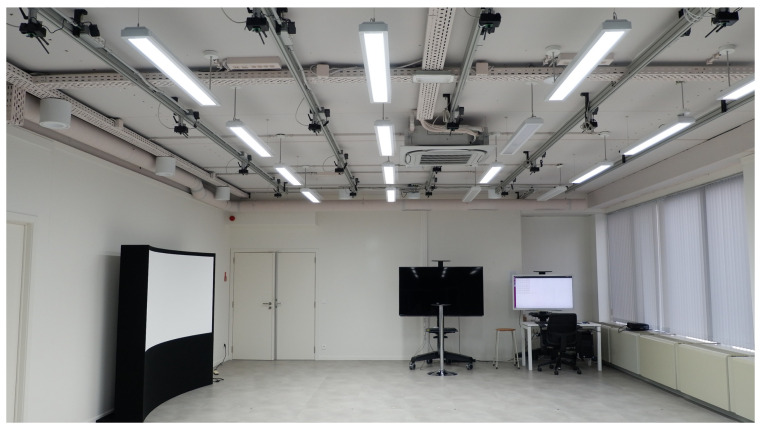
The proposed distributed VSN system, with 16 raspberry-pi cameras on the ceiling and a central service on the computer.

In our prior work, we proposed a novel approach for robot tracking based on 1D barcode localization and identification [8]. Due to the lack of appropriate triggering mechanisms and a comprehensive transmission protocol, the method suffered from low frame rates when the tracker was deployed on low-power embedded devices. To achieve both robust and real-time barcode tracking in a low-power VSN, we presented a distributed visual processing system via substantial algorithmic changes in collaborative barcode localization [9]. In this paper, the first comprehensive system of distributed VSN is presented as an extension of the work in [9]-see Figure 1. The proposed distributed VSN system is capable of performing real-time multi-target localization, tracking, and robust target identification based on barcodes. The contributions of the proposed system are listed as follows:

1. In contrast to the centralized coordination for barcode tracking in [8], we propose a completely distributed system with collaborative processing among nodes based on the proposed dynamic triggering mechanism and the hierarchical transmission protocol. Moreover, the server is only used to display the processed information of barcode tracking and localization from the VSN.
2. We propose a dynamic triggering mechanism to ensure that the visual sensor nodes work collaboratively. The information concerning incoming and outgoing barcodes is transmitted among neighboring nodes to schedule the activation states of nodes in the network. The dynamic triggering mechanism significantly decreases the consumption of energy and bandwidth, as well as improving the accuracy of barcode tracking and localization.
3. Looking at previous work [9], we designed a hierarchical scheme of transmission protocols to separately transmit the video and message data. In this scheme, multiple communication protocols are employed by the different components for different types of data. Specifically, the Real-Time Transport Protocol (RTP) [10] is used for video communication, while both the Transmission Control Protocol (TCP) and Long-Range (LoRa) protocol [11] are ultilized for message passing.

Furthermore, we provide a more in-depth analysis of the proposed algorithms, as well as a more rigorous mathematical formulation that allows for an objective evaluation of the proposed distributed framework. We demonstrate, through extensive experiments, that the real-time distributed VSN based barcode tracking and localization brings robustness with mm-level accuracy on the ground, as well as the rate and the energy savings, compared to an equivalent system performing centralized processing.

## 2. Related Work

Alternative solutions for indoor localization and asset tracking include radio-based solutions, based on BLE [12], UWB [13], and WIFI [14]. Those approaches face particularly difficult challenges in real-world logistics/production environments due to reflections of radio waves with numerous metallic objects, multi-path propagation, lack of perfect synchronization between the fixed anchors, and so on. The mean location errors obtained in real-world environments with radio-based solutions are spatially variable, and so are the standard deviations of the location errors. The authors are not aware of existing methods providing systematic location accuracy of less than 10 cm with radio-based solutions. Even if perfectly calibrated, the inherent changes in the monitored environment (moving merchandise, people, robots, etc) incur multi-path propagation, which in turn, alters the location accuracy in an unpredictable manner. In this sense, the proposed method serves as powerful alternative, providing accurate location of tracked assets irrespective of the dynamics in the monitored environment.

Robot localization can be achieved using a wide range of sensing hardware, such as infrared sensors [15,16], ultrasonic sensors [17], laser rangefinders [18] and RFID readers [19]. The state-of-the-art in real-time, camera-based localization solutions (ARTTrack5, 2017; Vicon Object Tracker, 2017) have subpixel accuracy while capturing at a frame rate of up to 420 Hz. However, those systems are exceedingly expensive for the area they cover and do not scale well [20] (e.g., ARTTrack5 is limited to 50 cameras per system, covering about 100 m). In contrast, this paper proposes a low-cost distributed visual sensor networks for the real-time localization and identification of multiple barcodes.

Barcode detection and tracking in video has been proposed in [8]. The work in [8] demonstrated that it provides a potential solution for robot localization and tracking with distributed VSN. Barcode detection is a well-researched area. Many approaches are proposed based on blob detector [21], bottom-hat filter [22], and mathematical morphology [23]. In centralized or distributed VSN, the cooperative information from the neighboring nodes improves the accuracy of barcode localization and identification. However, it is challenging to establish a real-time system with low-power cost for barcode localization and identification.

A typical VSN topology consists of a centralized architecture where all sensor nodes communicate through a central sink node [4]. However, the obvious weakness of such a centralized architecture is the vulnerability of the system, where the central sink node orchestrates all communication between the other nodes. Attempts have been made to alleviate this problem by introducing optimized multi-hop communication schemes [24] or error-correction schemes [25,26]. The fundamental idea is to reduce the message payload via reducing the resolution of the images. Even though the vulnerability is mitigated with the constraint of transmitted data, the centralized system still suffers from latency induced by the superfluous routing through the central node [27].

In [1,28], the centralized based routing is replaced with distributed sensor network systems, achieving more robustness and lower congestion of the overall system. In [1], a distributed sensor network-based surveillance system is described, which enables interaction between any two neighboring nodes. A variety of distributed VSN have been proposed for applications such as target detection [16], autonomous parking [17] and robot localization [15].

In distributed systems, direct communication is performed amongst the neighboring nodes. However, signal processing and information analysis are still executed independently [29]. Distributed analysis methods were proposed, which collaboratively process information coming from neighboring nodes [30,31] to accomplish object tracking and localization. A distributed object tracking algorithm is described in [32], where the target’s position is estimated with high accuracy. In [33], a distributed Kalman–Consensus filter is presented, which reaches a consensus with neighboring cameras about the status of tracked targets. A distributed negotiation strategy is described in [34] to achieve the best consensus state for the network within multiple tracked targets. In [5], a cubature information-filter-based distributed analysis method is presented for object-tracking in VSN. Inspired by the distributed analysis method for target tracking, we proposed a distributed barcode tracking method on Kalman filter in prior work [9]. In this paper, we propose an accomplished system performing distributed barcode tracking based on a novel dynamic triggering mechanism and a hierarchical transmission protocol.

The collaborative processing methods significantly improve the accuracy of target localization and tracking. However, the transmission among the nodes causes increased energy and bandwidth consumption. In operational conditions requiring limited bandwidth and energy consumption, the triggering mechanism has become an efficient way to address the problem of overload for the distributed system, allowing the selected cameras which contain informative content to be activated. In [35], an energy-efficient adaptive sensor scheduling strategy is presented, which selects the tasking nodes. Compared to a non-adaptive scheduling mechanism, the method enables the optimization to achieve the best tradeoff between the energy consumption and the predicted accuracy. However, the adaptive scheduling method has difficulties in predicting accurate states for multiple nodes and targets. Furthermore, [36] proposes a sleep scheduling mechanism to increase the energy efficiency with limitations on the tracking accuracy. The distributed object-tracking method of [5] provides a triggering mechanism to schedule the states of a node, i.e., active or sleeping, by measuring if a node’s informative content is beyond a threshold. Afterwards, Liu presents a multi-sensor scheduling approach based on the adaptive dynamic programming algorithm for cooperative target tracking [6]. However, the triggering mechanism in prior work [5,6] is often operated in a centralized way, fusing the information of multiple nodes in a sink node to predict the states. In this paper, we propose a dynamic triggering method that is deliberately designed to operate in a fully distributed manner.

Since the transmission is carried out in a distributed manner, a hierarchical transmission protocol is presented to deal with the multiple types of data. Our prior work [8] utilized the LoRa protocol [11] to transmit the data, which has the advantage of long-range coverage. However, the LoRa protocol is unable to transmit a large amount of video data within the low power VSN. Thus, the work in [8] is impaired by the extremely low frame rate, less than one frame per second. In [7], multiple types of data are packed and then transmitted via a single Ethernet protocol. Since the package combines multiple types of data, possible package losses could cause severe target tracking problems in the distributed VSN. Therefore, a hierarchical transmission protocol is first presented to alleviate the damages incurred by package losses by separately transmitting data depending on type. With this respect, the syntax elements of multiple types of message are given in the protocol. This combines the advantages of the RTP and the LoRa to achieve a high transmission efficiency.

## 3. Proposed Distributed Visual Sensor Network Architecture

**Figure 2:**
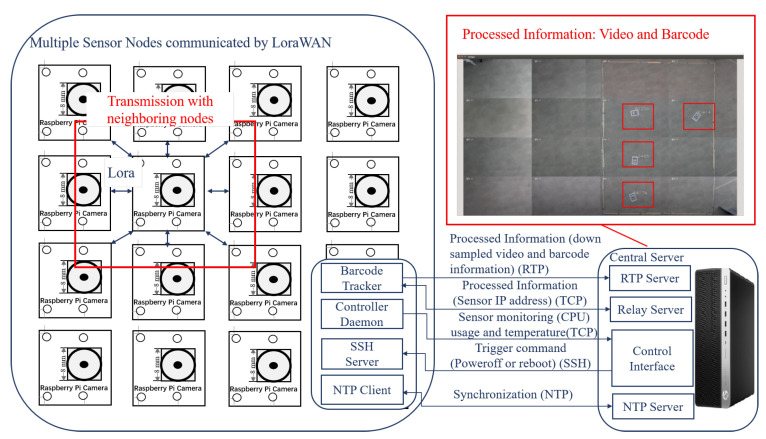
Overview of the distributed VSN system:16 Raspberry-pi cameras connected via LoRa, cameras and a central server is connected via RTP, TCP, SSH, and NTP.

As illustrated in Figure 2, we first present a real-time distributed VSN system to perform multi-barcode localization, tracking and identification. Specifically, the distributed barcode localization is operated in a predefined area, which is determined based on the location information received from neighboring node. Simultaneously, the identification algorithm decodes the barcodes into digit numbers. The processed information consists of the monitored video and the barcode’s location labelled as red rectangle. (see in Figure 2’s red box).

The system employs low-cost Raspberry-pi cameras as sensors; their state is scheduled by the proposed dynamic triggering mechanism. The fundamental idea of the proposed triggering mechanism is that the nodes are activated/deactivated depending on whether there are barcodes in their field of view. The triggering mechanism consists of an information exchange workflow, and the triggering condition, as further detailed in Section 3.2. The basis of the triggering mechanism is enabled by the information exchange among nodes provided by the proposed transmission protocol.

Comparing to previous work [9], we propose a hierarchical transmission protocol for four types of information. The information exchange within the proposed VSN architecture contains four cases shown in Figure 2: (1) processed information, which includes downsampled videos from cameras, barcode localization and identification status, and relay message among nodes; (2) sensor-monitoring information containing sensor’s CPU usage and temperature; (3) trigger commands for powering off, rebooting and updating nodes; (4) synchronization information used to synchronize the system clocks from all the sensors and the central server over NTP. The transmission among the nodes are via the LoRa protocol (see in Figure 2), and the exchange between the server and the nodes is based on RTP, TCP, SSH, and NTP. Next, we detail the syntax elements for multiple types of messages.

The remainder of the section is organized as follows: The distributed barcodes’ localization and identification are detailed in Section 3.1. The novel aspects brought in the proposed distributed platform include the dynamic triggering mechanism presented in Section 3.2 and the hierarchical transmission protocol described in Section 3.3.

### 3.1. Distributed Barcode Localization, Tracking and Identification

The proposed method performs multiple robot tracking, with the main algorithms including barcode localization and identification in videos. The distributed barcode-tracking scheme announces incoming barcodes to neighboring nodes. Furthermore, barcode localization is operated in a predefined area, which is determined based on the location information received from neighboring nodes. Afterwards, the barcode extraction algorithm is presented to extract the rectified barcode from an image. Moreover, the identification algorithm decodes the bars in searched barcode into digit numbers according to the width of each bar. The algorithm performing barcode localization, tracking, and identification is explained in detail in the following.

#### 3.1.1. Barcode Location Prediction

The prediction process consists of two steps: barcode location prediction and location correction. First, the locator is used to localize the barcode based on the predicted location, whereby a predictor is used to estimate the location of each barcode in each node. After that, the prediction is corrected using the newly acquired location information. In contrast to the centralized Kalman filter employed in [8], in this work, we propose a distributed prediction method, which allows each node to employ its own predictor, such as Kalman filter [37], invariant Kalman filter [38], or Square-root unscented Kalman filter (SRUKF) [39]. Theoretically, the improved versions of Kalman filtering perform better than the original Kalman filter, but the complexity is also higher. However, to maintain a balance between accuracy and computational complexity, we leverage the classical Kalman filter as the predictor employed in the proposed distributed barcode-tracking system. The reason for this is that the novel dynamic triggering mechanism allows the tracker to search in small areas instead of searching the entire frame, based on the previous locations of barcodes. In addition, as shown in the experimental results in Section 4.1, the proposed distributed prediction method based on Kalman filtering has reached a less than 1 cm location error in cases of high and low bit costs (covering a relatively-wide QP range from 12 to 37). This proved to be sufficient to perform real-time barcode tracking with the proposed system.

In principle, further improvements in the location accuracy of the estimated trajectories and locations are expected to be obtained by improved versions of Kalman filtering [38,39]. These filtering techniques have the potential to provide more stable location predictions at lower resolutions and higher QPs but will come with additional computational complexity. Investigating improvements in the proposed method based on improved Kalman filtering techniques and assessing the performance-complexity trade-offs are left as topics of further investigation.

#### 3.1.2. Barcode Locator

**Figure 3:**
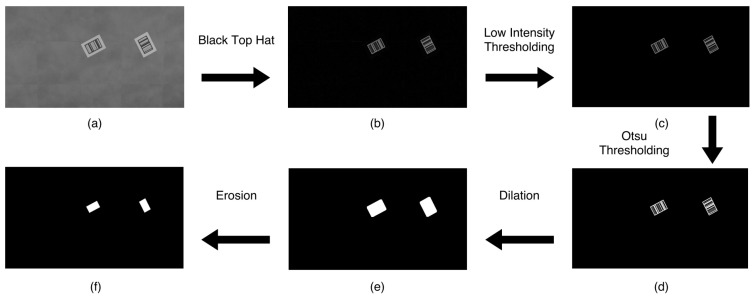
Barcode detection: (**a**) grayscale input image, (**b**) result after black top-hat transform, (**c**) result after low-intensity thresholding, (**d**) result after Otsu thresholding, (**e**) result after dilation, (**f**) result after erosion and final result.

After the barcode’s location is predicted, the barcode localization is continued with two steps: the barcode detection inspired by the work of [23] and the refinement of the detection. As depicted in Figure 3, the procedures are listed as follows:

- A black top-hat (i.e., bottom-hat) transform is applied to emphasize the white bars of the barcode and produce a highly contrasted image.
- Low-intensity pixels are removed to improve performance in the next step.
- A binary image is produced by means of automatic thresholding with Otsu’s method [40].
- The binary image is dilated to expand the barcode regions.
- A final erosion step trims the foreground regions and removes areas that are too small to actually be part of a barcode.

**Figure 4:**
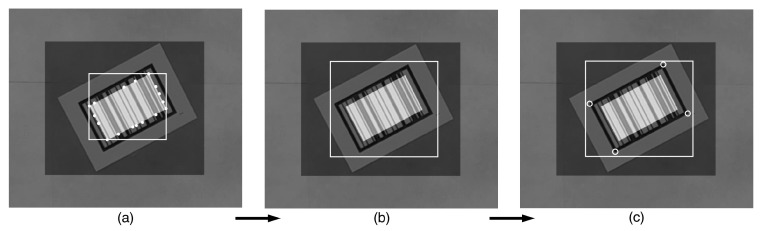
Bounding-box algorithms for refining localization: (**a**) finding the bounding box, (**b**) scaling the bounding box, (**c**) detecting four strong corners.

The result should be an image that delineates the position of the barcode. The refinement step illustrated in Figure 4 consists of the following steps:

- Finding the bounding box of the detected barcode.
- Expanding this bounding box so that it includes the entire barcode.
- Detecting the four corners of the barcode border. We employ Harris corner detection to locate the barcode corners.

#### 3.1.3. Barcode Extraction

**Figure 5:**
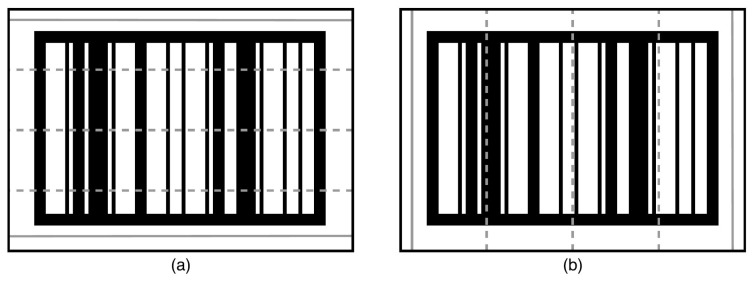
Diagram showing the sample lines that are extracted from images potentially containing barcodes. (**a**) five horizontal sample lines, (**b**) five vertical sample lines.

**Figure 6:**
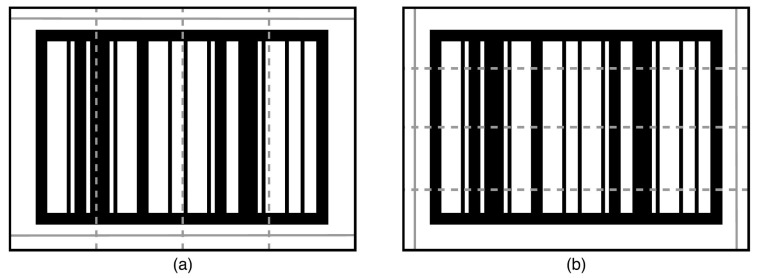
Diagram showing the two combinations of sample lines. (**a**) two horizontal and three vertical sample lines, (**b**) three horizontal and two vertical sample lines.

Once that the barcodes are successfully located, the extraction algorithm is utilized to extract the barcode from the located area for identification. The extraction process uses the points of the four corners (shown in Figure 4c) provided by the locator and applies a perspective transform on the input images based on those four points. The result of that process is an image containing only the rectified barcode. From that rectified barcode image, the barcode extraction algorithm aims to (i) determine whether the input image contains a barcode, and (ii) extract barcodes from the image. Knowing if an image contains a barcode is an important aspect. Eliminating potential locations that do not contain a barcode is essential for ensuring the real-time performance of the tracking algorithm. From that rectified barcode image, five horizontal and five vertical sample lines are extracted to determine the barcode orientation, as shown in Figure 5; the combination of horizontal and vertical sample lines is illustrated in Figure 6.

The first step is to determine the number of black-white/white-black transitions along the sample lines, formulated in Equation (1):

(1) $$R_{i}=E_{i}/P_{i},$$

where $R_{i}$ denotes the ratio of transition, $E_{i}$ is the number of transitions, and $P_{i}$ the number of pixels in line *i*. Secondly, the ratio $R_{i}$ is used as input to determine the orientation of the barcode according to the rules, such as:

- The orientation is horizontal if the function returns none for the first combination in Figure 6a and horizontal for the second combination in Figure 6b.
- The orientation is vertical if the function returns vertical for the first combination in Figure 6a and none for the second combination in Figure 6b.

Finally, the decoding of the barcode is fulfilled on the extracted barcode including information on the position, the orientation, and the rectified barcode.

#### 3.1.4. Barcode Identification

**Figure 7:**
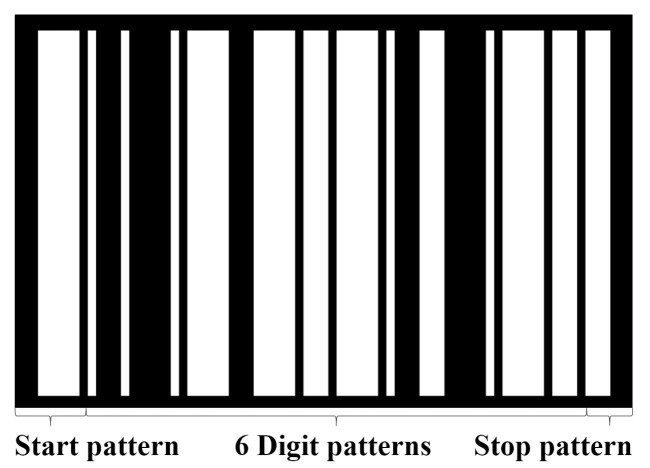
Barcode structure and example. Encoded number: 12465.

The identification algorithm assigns a number for each digit pattern within a barcode. In Figure 7, the barcode starts with a start pattern, followed by six digit patterns, and a stop pattern. Out of the six patterns, five patterns are used to encode the actual number. All numbers are ranged in [00000–77,777], which are equivalent to [0–32,767] in decimal notation. The sixth digit is used for error detection and is calculated as Equation (2):

(2) $$D_{6}=D_{1}⊕D_{2}⊕D_{3}⊕D_{4}⊕D_{5},$$

where $D_{i}$, i∈[1,6] is the ith digit, and ⊕ is the bitwise XOR operator. A digit pattern is the combination of four white and black bars, where the width of each bar is different.

For instance, in Figure 7, the first digit pattern corresponds to a combination of two white lines and two black bars, where the width of bars is (white, black, white, black) =(1,3,1,5). According to the designed rule of digit pattern listed in Table 1, the combination of (white, black, white, black) =(1,3,1,5) corresponds to the number 1.

Once the 5 digits are decoded, the sixth digit is calculated for error detection using Equation (2). Additionally, the decoder calculates a confidence about the decoded barcode. The confidence number can range from 0.0 (not decoded at all) to 1.0 (fully decoded).

### 3.2. Dynamic Triggering Mechanism Amongst Sensors

**Figure 8:**
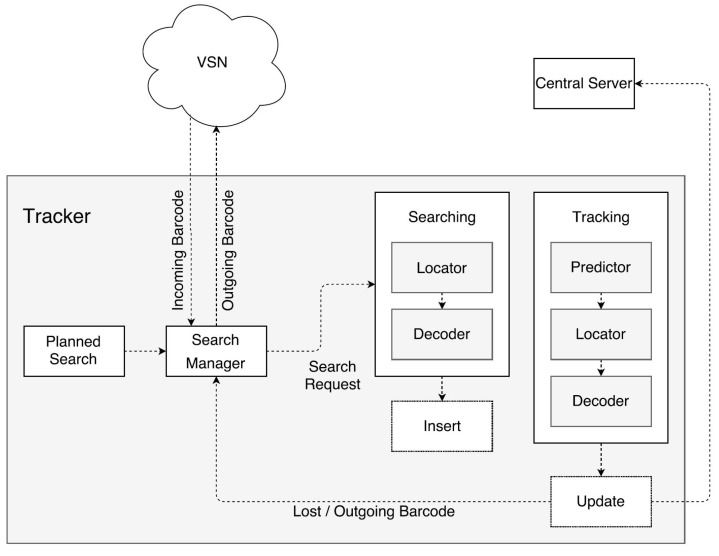
Workflow of barcode tracking instance.

The proposed dynamic triggering mechanism schedules the activation states for each node. During the barcodes’ tracking, the information involving the incoming and outgoing barcode is transmitted among neighboring nodes. In Figure 8, the rule of scheduling states that whenever a node detects a barcode, it sends the tracking information to neighboring nodes. A tracker can receive barcode information at any point in time.

In the diagram (see Figure 8), there are four main components in tracking barcodes. The first component is the Search Manager, which acts when the tracker is activated. The Search Manager receives a planned search, which creates multiple search requests for different regions of a frame. Those search requests are stored in a list and can be either time-limited or for one-time use only. The next component to start working is the tracker itself, which takes *n*_s_ search requests from the Search Manager and creates search jobs for them. The value of *n*_s_ depends on the number of free jobs *n*_free_ per frame. Once all jobs are completed, the tracker will add all newly found barcodes to a list for further tracking. These tracked barcodes will also become jobs, called track jobs, when the next frame is being processed. The number of track jobs *n*_t_ is not limited at all. It only limits *n*_s_ in the following way. Let *n*_tot_ be the advised maximum number of jobs; then, *n*_free_ = *n*_tot_ − *n*_t_. In other words, less important search jobs cannot fill the tracker when it is executing more useful track jobs, formulated in Equation (3) as:

(3) $$0\leq n_{s}\leq n_{\mathrm{free}}\leq n_{\mathrm{tot}}.$$

At the searching component, there are two steps. The first step, the Locator, is responsible for determining the precise locations of barcodes within images, as detailed in Section 3.1.2. The second step, the Decoder, is responsible for decoding the barcodes that were detected during the previous step, as explained in Section 3.1.4.

The last component is the tracking process. The first step of the tracking component is the predictor. The predictor predicts the future locations of barcodes based on their previous locations, as detailed in Section 3.1.1. The remaining steps in the tracking process are the same components as in the searching component.

The main difference between tracking and searching is the way in which new information is processed. After searching, only the newly found barcodes can be inserted and the barcodes that were marked lost can be updated. Barcodes that had already been tracked are not updated during this step because a track job is already running for those barcodes. If a search job would update a tracked barcode, it ends up waiting for the track job to finish due to synchronization. Tracking, however, will never insert newly found barcodes. It is highly unlikely that a track job will actually find a new barcode.

**Figure 9:**
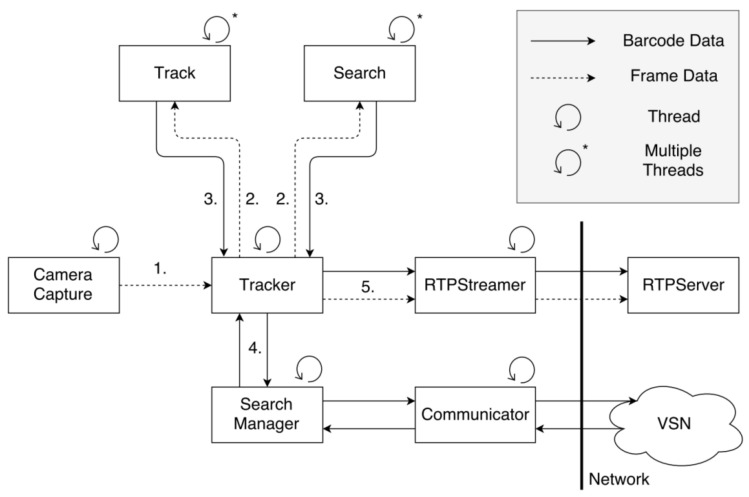
Workflow of barcode tracking instance.

The functioning of the searching and tracking algorithms is quite complex. Therefore, in Figure 9, we illustrate the information flow of the barcode tracking performed in the proposed distributed VSN. The arrowheads indicate the direction in which information is sent between the different modules of the barcode tracker. The type of arrow indicates the type of data that are communicated (dashed: frame data; full-line: barcode data). The numbers next to the arrows indicate the order in which the data are sent.

- In a first step, a frame is captured by the camera and sent to the Tracker module (arrow 1).
- The Tracker then creates search and track threads depending on the number of already tracked barcodes and pending search requests (arrow 2).
- These threads execute the track and search algorithms. The search for a barcode is performed in a predefined area, determined based on the information received from the barcode trackers running on the neighboring cameras in the VSN. The tracking algorithm first predicts the location of the tracked barcode.
- Then, it attempts to localize the barcode in an area defined around the predicted location. When these threads finish executing, they send the processed information back to the Tracker (arrow 3).
- The tracker then processes that information to see if there are outgoing barcodes. If so, the information is sent to the Search Manager (arrow 4), which manages incoming and outgoing search requests and wakes up the node that is likely to see the outgoing barcode.
- Next, the Tracker sends the frame data, together with the processed information for that frame, to the RTP Streamer (arrow 5). That data are then sent to the central server for visualization.
- The data from the different nodes are synchronized by the central server using timestamps. The system clocks of all nodes are synchronized with the system clock of the central server so that the maximum difference between any two nodes is 2 ms.

### 3.3. Hierarchical Transmission Protocol

#### 3.3.1. Communication between Sensors and Server

The central server consists of four components as shown in Figure 2. The first component, the RTP Server, acts as a receiver for the processed information, which is sent from the VSN using an RTP session. The most important job of the RTP Server is to synchronize all the incoming data. The RTP Server receives frame data together with barcode information for that frame.

**Figure 10:**
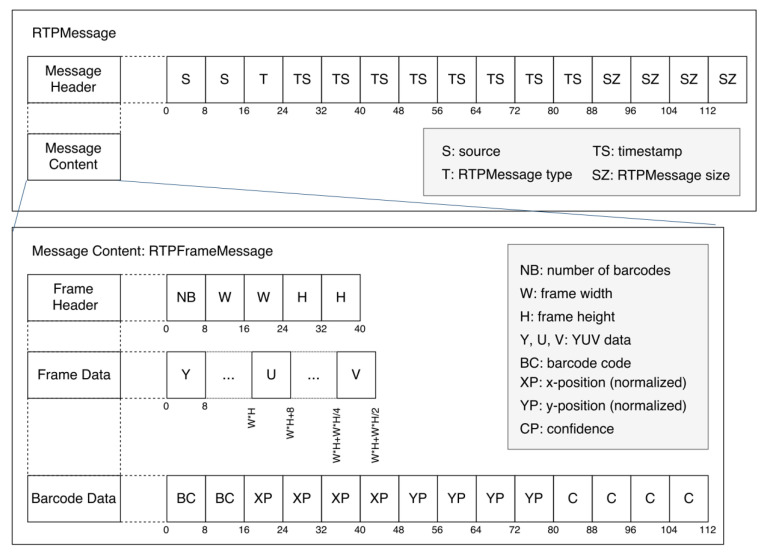
RTP message structure composed of a message header and content.

Syntax element: The RTP message structure is composed of a message header and content, as illustrated in Figure 10. The message header contains the ID number of the sender, message type, the timestamp of the message, and message size. The message content consists of a frame header, frame data, the barcode code, and barcode location. This information is synchronized and then displayed to the user. The right hand side of Figure 2 shows a screenshot of the output produced by the RTP Server. The server provides a live display of the sixteen video streams coming from the cameras and the corresponding barcode information.

The second component, the Relay Server, relays messages from one node to another and is built using low-level system calls to optimize performance. The server requires all nodes to send a simple login message, using TCP, before they can receive messages from another node. The server requires all nodes to send a simple login message before they can receive messages from another node.

**Figure 11:**
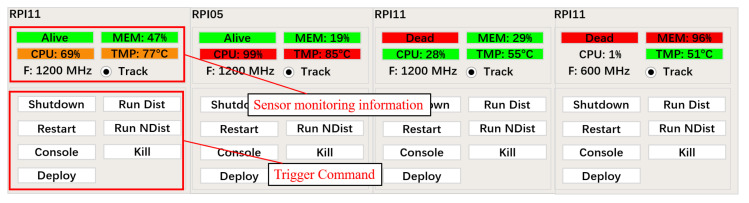
Control interface: sensor monitoring and trigger command.

The third component, the Control Interface, visualizes the VSN. This is a basic Graphical User Interface (GUI) that allows for monitoring and control over the VSN. The first role of the Control Interface is to display information concerning the status of the nodes in the VSN. Typical status information consists of the Central Processing Unit (CPU) usage, CPU temperature, CPU frequency, and main memory usage. Figure 11 shows four different health states of a node:

- High CPU usage and temperature.
- Very high CPU occupancy and temperature, causing thermal throttling.
- A node that was once powered on but got powered off or is not responding,
- memory leak.
- A node that is not responding due to a large memory leak or very high memory usage.

Without this GUI, these problems would be very difficult to detect. The second role of the Control Interface is to control the different nodes in the network, deploy updates of the tracker software and control the tracker software. These different commands are transmitted from the central server to the sensor nodesthrough SSH.

The last component inside the central server is an NTP server. The NTP server is used by all the nodes in the VSN to synchronize their system clocks to the system clock of the server.

#### 3.3.2. Communication among Sensors

The overall efficiency of the distributed VSN relies on the efficient communication between the sensor nodes. The basic idea behind the proposed distributed tracking system is that the nodes in the VSN can go into sleep mode if no barcode appears in their field of view. Neighboring nodes will wake up a node whenever a barcode is likely to enter that node’s field of view. The distributed network saves energy compared to a VSN with independently operating cameras, whereby each node searches for barcodes, tracks and decodes them at all times. Collaborative processing of tracking information is a crucial component of the proposed distributed VSN.

**Figure 12:**
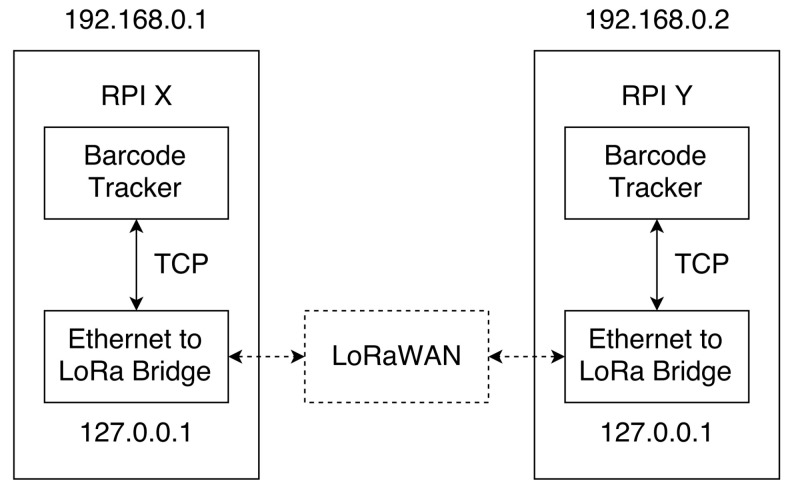
Communication mechanism between sensors over LoRaWAN.

Communication between the nodes is achieved over LoRaWAN (as depicted in Figure 12), which represents a low-power, wide-area network protocol deployed on the VSN. More specifically, the barcode tracker is connected to an Ethernet to LoRa Bridge and the message is transmitted via LoRaWAN. To enable future extension, the bridge has the same property as a regular TCP connection.

**Figure 13:**
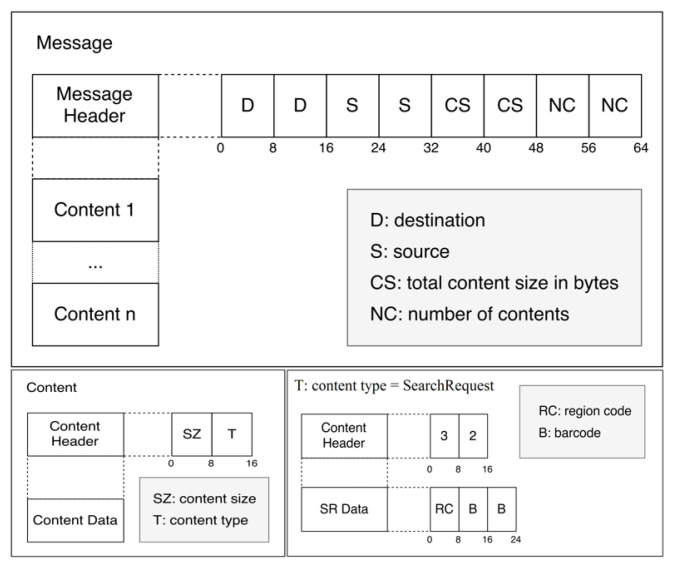
Diagram showing the structure of messages and the content of those messages.

Syntax element: A typical message is composed of header and content, as shown in Figure 13. The message header indicates the destination node, source, content size in bytes, and the number of content sections. The first part of a content section is the header, which contains the content section size in bytes and the type of content. The other part of the content section is only content data. Figure 13 also contains an example of a content section. By allowing for different content types, communication becomes versatile. Efficient communication is achieved by allowing a message to contain multiple content sections.

## 4. Experimental Results

The proposed system is composed of a server with Intel(R) Xeon(R) CPU E5-1650v3 at 3.50 GHz, with 64 GB of RAM, and a low-power VSN with 16 Raspberry Pi 3 (RPI) nodes, arranged in a four by four grid, as shown in Figure 1. All sensors are equipped with a full HD 1920×1080 camera and connected in an Ethernet star-shaped network. The area covered by one camera is about 3 m${}^{2}$. There is slight overlap on the boundary of the areas. We design the system in an efficient, scalable and flexible way. The RPI nodes use a micro-SD card as secondary storage, which is limited in size and speed compared to a regular hard-disk drive or solid state drive. This has the unfortunate side effect that it is not possible to store the captured frames while tracking. However, it is possible to store compressed frames when the tracker is not running.

### 4.1. Barcode Miss Rate and Localization Error

Due to bandwidth constraints and the limited write speed of the SD-card on the nodes, the input video has to be compressed. Compression artefacts and down-sampling are two critical factors that affect the accuracy and robustness of the barcode localization and identification. Thus, we evaluate the impact on the performance of barcode localization and identification at different compression ratios and downscaling rates. The compression ratio is is controlled by the quantization parameter (QP) of the video codec.

**Figure 14:**
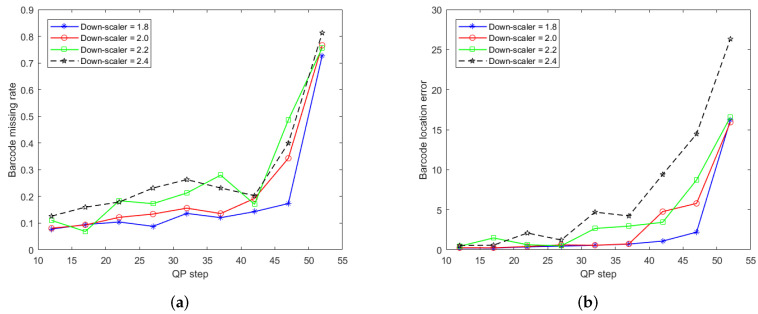
(**a**) Barcode’s missing rate (%) and (**b**) barcode’s location error (cm) against QP and down scaler.

Sixteen videos are captured and tested in the experiments. The average barcode miss rate and location errors are listed in Table 2 and Table 3 and the corresponding plots are illustated in Figure 14a,b, respectively.

The barcode missing rate $I_{r}\left(s,q\right)$ is calculated as the percentage of failed barcode identifications at the quantization step *q* and the downscaling factor *s*, expressed in Equation (4),

(4) $$I_{r}\left(s,q\right)=\frac{N_{f}\left(s,q\right)}{N_{W}},$$

where $N_{f}\left(s,q\right)$ is the number of frames that barcode identification was unsuccessful and $N_{W}$ is the total number of frames in the video.

The other factor that expresses barcode localization accuracy is the location error h(s,q) (centimeter, cm), measured by the Euclidean distance between the prediction location via our system and the real location. To relate real-space coordinates with coordinates in the captured video, a grid is demarcated on the floor in our experimental scene. We employ the symbol *c* to denote the ratio between the real-space coordinate and the coordinate in the video. The formulation of h(s,q) is expressed in Equation (5),

(5) $$h\left(s,q\right)=E_{d}\left(c\times V\left(s,q\right);R\left(s,q\right)\right),$$

where $E_{d}\left(:;:\right)$ represents the Euclidean distance between two coordinates, and V(s,q) and R(s,q) are the predicted coordinate in the video and the real-space coordinate, respectively, expressed in centimeter (cm).

We can observe from the plots in Figure 14 that the proposed distributed method performs well for QPs in the range QP∈[10,42]. The barcode missing rate $I_{r}$ and barcode localization error $L_{e}$ vary in the range $I_{r}\in \left[0.08,0.28\right]$ and $L_{e}\in \left[0.23,9.4\right]$. Beyond a QP step of 42, the barcode miss rate and location error significantly increase. The experimental results demonstrate that the proposed barcode localization and identification algorithms are robust for appropriate QP settings.

The experiments at different downscaling ratios show that the more the size of the frame is reduced, the larger the localization error becomes. In the case of a downsampling ratio of s=1.8, the miss rate is below 0.1 within the range QP∈[10,42]. For higher downscaling factors s∈[2.0,2.4], the miss rate becomes larger than 0.1. A larger downscaling ratio results in a higher localization error, as can be observed in Figure 14.

### 4.2. Bandwidth Consumption

In the proposed VSN architecture for the tracking and identification of multiple barcodes, the streaming size $B(x_{i},t_{i})$ of node i∈[1,16] is proportional to the size of the message that contains the barcode digits and its location, transmitted via LoRaWAN with a bitrate of $\alpha_{i}$, and the video stream to be visualized in the central server with bitrate $\beta_{i}$ in Equation (6).We denote the payload of the video stream and message information of node i∈[1,16] as $x_{i}$. Then:

(6) $$B\left(x_{i},t_{i}\right)=\frac{\alpha_{i}\times {t_{i}}^{\prime }+\beta_{i}\times t_{i}}{t_{i}},$$

where ${t_{i}}^{\prime }$ is the time spent on transmitting the message with barcode digits and coordinates, $t_{i}$ is the time period that $x_{i}$ is transmitting and $B(x_{i},t_{i})$ is the size in bits of the data stream transmitted during the time period $t_{i}$.

We define the bitrate of centralized network $R^{\prime }$ in Equation (7),

(7) $$R^{\prime }=16\times \frac{\sum_{i=S(1)}^{S(N)}B\left(x_{i},t_{i}\right)}{\sum_{i=S(1)}^{S(N)}t_{i}}.$$

Moreover, the total bitrate *R* of the distributed network is given by Equation (8),

(8) $$R=\frac{\sum_{i=S(1)}^{S(N)}B\left(x_{i},t_{i}\right)}{\sum_{i=S(1)}^{S(N)}t_{i}}.$$

Since ${t_{i}}^{\prime }\ll t_{i}$, the streaming size can be considered $B\left(x_{i},t_{i}\right)\dot{=}\beta_{i}$, leading to a bitrate saving of $R_{saving}=\left(R^{\prime }-R\right)$.

Bandwidth consumption is proportional to the amount of video streams transmitted from the visual sensors to the central server and data exchange among nodes. In the distributed system, the sensors are activated when there are stationary or moving barcodes in the area covered by the sensor. The corresponding videos of those barcodes are transmitted to the central server and messages are sent to neighboring nodes accordingly.

As a single object is moving in the covered area, it comes into the field of vision of a set of cameras *R*, where the number of activated cameras is *N*. For instance, if the barcode goes across cameras i=1 for $t_{1}$ seconds, camera i=2 for $t_{2}$ seconds, camera i=3 for $t_{3}$ seconds, the activated cameras’ set is S=[1,2,3] and N=3.

**Figure 15:**
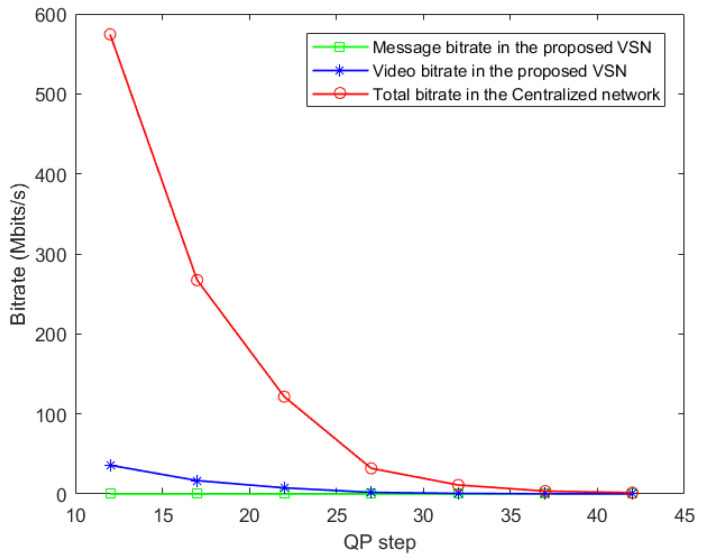
Average bitrate cost against QP step.

The videos with a resolution of 1920×1080 are compressed and then transmitted from the nodes to the central server with multiple QP values. Table 4 reports the cost of message transmission via LoRa-based communication within 1 second against modulation bandwidth, spreading factor, and code rate. The bitrate comparisons are listed in Table 5 and the corresponding bitrate cost is plotted in Figure 15.

To compare distributed and centralized VSN at the same experimental conditions, we leverage the real centralized VSN system being implemented in our previous work [9]. In Table 5, a max bitrate of 35.9 Mbits/s at QP=12 is obtained for video transmission in the proposed distributed VSN, while the bitrate in the centralized VSN of [9] is 574.4 Mbits/s, i.e., 16 times that of distributed VSN. Experimental results indicate that the bitrate decreases with increasing QP value for both the distributed and centralized VSN.

For the message exchange between the nodes of the network, the bitrate consumption is 0.015 Mbits/s at 25fps per barcode. The message contains a header, the destination node, the source node and message body, consisting of the barcode number and its location. Since a message is transmitted when a barcode arrives at the edge area of the field of view, the bitrate is significantly smaller than the rate needed for video transmission. In our experiments, the bitrate for the transmitted video is from 2.9 to 4030 times smaller compared to the bitrate of the transmitted messages.

### 4.3. Power Consumption

The power estimate depends on the environment and its requirements. We estimate the power consumed by applying the proposed distributed VSN to a real-world warehouse hall, which is about 60 m × 120 m and has about 10 vehicles moving inside. A total of 276 sensors are needed to cover the entire warehouse. There are two power states that a sensor can have: sleeping or tracking. The power consumption of each node is denoted as $P_{sn}$ and $P_{tn}$ for sleeping and tracking state respectively, where $P_{sn}=1.4$ W and $P_{tn}=4.9$ W. The worst case for the entire system is that all ten vehicles are scattered around the warehouse, being seen by $N_{tn}=40$ different sensors. Each vehicle is seen by four neighboring sensors within their overlapping region.

We define the power of the worst case as $P_{w}$ in Equation (9),

(9) $$P_{w}=N_{tn}\times P_{tn}+\left(276-N_{tn}\right)\times P_{sn}.$$

Moreover, the power of the best case $P_{b}$ of the distributed network is given by Equation (10),

(10) $$P_{b}=276\times P_{sn}.$$

The best case is that the vehicles are not inside the field of view of any sensor (e.g., outside the warehouse or inside a lorry that is being loaded). We compare the power consumption between the worst and the best cases for the entire system in Table 6.

### 4.4. Time Complexity

The significant advantage of distributed tracking is that, when there are no barcodes in the frame, the distributed tracker does not waste time searching those barcodes. A centralized tracker has to search for new barcodes in every frame.

Three states can be represented in a sensor node: (i) a new barcode is entering the area it is responsible for, (ii) an already tracked barcode leaves the area or comes from a neighboring node, (iii) a decoded barcode is being tracked. The operations of each of these states are composed of basic processes: localization, decoding, prediction, and communication. Their duration of the basic processes are labelled $T_{l}\left(k\right),\;T_{d}\left(k\right),\;T_{p}\left(k\right),\;and\;T_{c}\left(k\right)$, respectively, where *k* corresponds to one of the three aforementioned states. Given the number of new barcodes $N_{p}$ and already tracked barcodes $N_{q}$, the duration of localization and decoding of a video frame is formulated in Equation (11):

(11) $$T_{\gamma }\left(k\right)=\left\{\begin{array}{c} t_{\gamma }\left(k\right)\times N_{p},k=1\\ t_{\gamma }\left(k\right)\times N_{q},k=2\\ t_{\gamma }\left(k\right)\times \frac{N_{p}+N_{q}}{\tau \times fps},k=3 \end{array}\right.,\gamma \in \left\{l,d\right\},$$

where $t_{l}\left(k\right)=t_{pre}\left(k\right)+t_{ref}\left(k\right)$, where $t_{pre}\left(k\right)$ and $t_{ref}\left(k\right)$ are the required times for pre-processing and refining a barcode, τ describes the waiting time between searches and fps is the framerate of the current video. The time required for decoding is defined as $t_{d}\left(k\right)=t_{ext}\left(k\right)+t_{dec}\left(k\right)$, where $t_{ext}\left(k\right)$ and $t_{dec}\left(k\right)$ are the time of extracting and decoding a barcode, respectively. For the third state, i.e., tracking a barcode, there is a periodic search of the entire frame. The time complexity for prediction is formulated in Equation (12):

(12) $$T_{p}\left(k\right)=\left\{\begin{array}{c} t_{pred}\left(k\right)\times N_{p},k=1\\ 0,k=2\\ 0,k=3 \end{array}\right.,$$

where $t_{pred}\left(k\right)$ is the time required for prediction. Finally, the time complexity for communication between two nodes is defined in Equation (13):

(13) $$T_{c}\left(k\right)=\left\{\begin{array}{c} 0,k=1\\ \left(t_{send}\left(k\right)+t_{rec}\left(k\right)\right)\times N_{q},k=2\\ 0,k=3 \end{array}\right.,$$

where $t_{send}\left(k\right)$ and $t_{rec}\left(k\right)$ denote the duration of sending and receiving a message containing location and barcode digits.

We define the expression for total time complexity as Equation (14):

(14) $$M_{dt}=\sum_{k=1}^{3}T_{sum}\left(k\right),$$

where

(15) $$T_{sum}\left(k\right)=T_{l}\left(k\right)+T_{d}\left(k\right)+T_{p}\left(k\right)+T_{c}\left(k\right),\;k\in \left[1,2,3\right].$$

**Figure 16:**
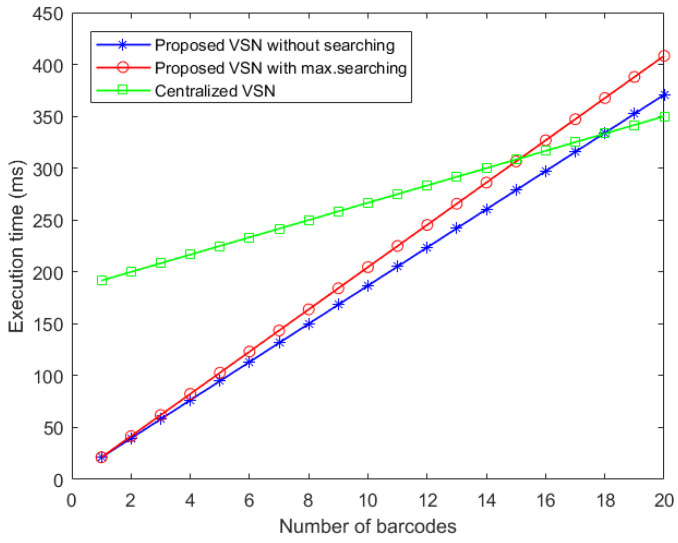
Execution time for the proposed and centralized camera tracking systems.

A comparison of the time complexity between a distributed and a centralized architecture is given in Figure 16. The x-axis represents the number of barcodes in a single camera’s field of view, while the y-axis corresponds to the execution time of a single barcode tracker executed on a single thread. The first observation is that there is a certain amount of barcodes above which the distributed tracker will be slower than the centralized counterpart, caused by the overhead per pixel in the distributed architecture. For the lower bound (performing maximum searching), this lies between fourteen and fifteen barcodes. For the upper bound, the intersection point lies between seventeen and eighteen barcodes. Clearly, this gain has an upper and lower bound depending on the number of searched barcodes. The upper limit will be reached when the number of searched barcodes is minimal. The lower limit will be reached when the number of searched barcodes is maximal.

## 5. Conclusions

This work proposes a low-power distributed visual-processing system to track multiple barcodes in real-time. A novel distributed tracking architecture was proposed and extensively evaluated under a practical testing environment in our lab. The architecture was designed to be efficient and to allow for future extensions. The proposed distributed VSN architecture incorporates a newly designed dynamic triggering mechanism and a hierarchical transmission protocol, facilitating efficient communication among neighbouring nodes. Experiments have shown that the distributed system is robust for barcode-based robot tracking and highly accurate for the localization, which is less than 1 cm error for an area of 3 m${}^{2}$ per camera. The proposed distributed system has the advantages of being energy-saving, and having scalable installation and low maintenance costs.

## Figures and Tables

**Table 1:** Barcode’s encoding rule of digit pattern.

Digit Pattern	Encoding	Length
start	3 5 1	9
0	1 5 1 3	9
1	1 3 1 5	9
2	1 1 5 3	9
3	3 1 3 3	9
4	5 1 3 1	9
5	3 5 1 1	9
6	5 1 1 3	9
7	5 3 1 1	9
stop	3 3	6

**Table 2:** Barcode missing rate $I_{r}$ (%) against QP and down scaler.

DOWN	QP								
SCALER	12	17	22	27	32	37	42	47	52
1.8	0.08	0.09	0.1	0.09	0.14	0.12	0.14	0.17	0.7
2.0	0.08	0.09	0.12	0.13	0.16	0.14	0.19	0.34	0.76
2.2	0.11	0.07	0.18	0.17	0.21	0.28	0.17	0.48	0.76
2.4	0.13	0.16	0.18	0.23	0.26	0.23	0.2	0.4	0.8

**Table 3:** Barcode localization error (cm) against QP and down scaler.

DOWN	QP								
SCALER	12	17	22	27	32	37	42	47	52
1.8	0.23	0.22	0.36	0.46	0.57	0.72	1.1	2.2	16.2
2.0	0.26	0.25	0.42	0.6	0.57	0.74	4.77	5.79	15.9
2.2	0.45	1.48	0.6	0.49	2.67	2.96	3.44	8.72	16.6
2.4	0.54	0.58	2.1	1.23	4.7	4.23	9.4	14.5	26.3

**Table 4:** LORA bitrates (code rate = 4/5).

Spreading	Chips	Modulation Bandwidth (BW)		
Factor	Symbol	125 KHz	250 KHz	500 KHz
6	64	9375 bps	18,750 bps	37,500 bps
7	128	5468 bps	10,937 bps	21,875 bps
8	256	3125 bps	6250 bps	12,500 bps
9	512	1757 bps	3515 bps	7031 bps
10	1024	976 bps	1953 bps	3906 bps
11	2048	537 bps	1074 bps	2148 bps
12	4096	292 bps	585 bps	1171 bps

**Table 5:** Average video bitrate (Mbits/s) against QP.

Method	QP Step						
	12	17	22	27	32	37	42
Proposed DVSN	35.9	16.7	7.6	2	0.7	0.22	0.1
Centralized VSN [9]	574.4	267.2	121.6	32	11.2	3.5	1.6

**Table 6:** Comparison of power consumption between the worst and the best case for the entire system.

Power Consumption of Multiple States	Sleeping	Tracking	Overall Power
power per sensor	1.4 W	4.9 W	
Worst case VSN	236 sensors	40 sensors	526.4 W
Best case VSN	276 sensors	0 sensors	386.4 W
No sleep VSN	0 sensors	276 sensors	1352.4 W

## Data Availability

Not applicable.
